# Development and application of specific FISH probes for karyotyping *Psathyrostachys huashanica* chromosomes

**DOI:** 10.1186/s12864-022-08516-6

**Published:** 2022-04-18

**Authors:** Hao Zhang, Fei Wang, Chunyan Zeng, Wei Zhu, Lili Xu, Yi Wang, Jian Zeng, Xing Fan, Lina Sha, Dandan Wu, Yiran Cheng, Haiqin Zhang, Guoyue Chen, Yonghong Zhou, Houyang Kang

**Affiliations:** 1 State Key Laboratory of Crop Gene Exploration and Utilization in Southwest China, Sichuan Agricultural University, 611130 Chengdu, Sichuan China; 2 Triticeae Research Institute, Sichuan Agricultural University, 611130 Chengdu, Sichuan China; 3 College of Resources, Sichuan Agricultural University, 611130 Chengdu, Sichuan China; 4 College of Grassland Science and Technology, Sichuan Agricultural University, 611130 Chengdu, Sichuan China

**Keywords:** Fluorescence *in situ* hybridization karyotype, *Psathyrostachys huashanica*, FISH painting, Chromosome identification, Addition lines

## Abstract

**Background:**

*Psathyrostachys huashanica* Keng has long been used as a genetic resource for improving wheat cultivar because of its genes mediating the resistance to various diseases (stripe rust, leaf rust, take-all, and powdery mildew) as well as its desirable agronomic traits. However, a high-resolution fluorescence *in situ* hybridization (FISH) karyotype of *P. huashanica* remains unavailable.

**Results:**

To develop chromosome-specific FISH markers for *P. huashanica*, repetitive sequences, including pSc119.2, pTa535, pTa713, pAs1, (AAC)_5_, (CTT)_12_, pSc200, pTa71A-2, and Oligo-44 were used for a FISH analysis. The results indicated that the combination of pSc200, pTa71A-2 and Oligo-44 probes can clearly identify all Ns genomic chromosomes in the two *P. huashanica* germplasms. The homoeologous relationships between individual *P. huashanica* chromosomes and common wheat chromosomes were clarified by FISH painting. Marker validation analyses revealed that the combination of pSc200, pTa71A-2, and Oligo-44 for a FISH analysis can distinguish the *P. huashanica* Ns-genome chromosomes from wheat chromosomes, as well as all chromosomes (except 4Ns) from the chromosomes of diploid wheat relatives carrying St, E, V, I, P and R genomes. Additionally, the probes were applicable for discriminating between the *P. huashanica* Ns-genome chromosomes in all homologous groups and the corresponding chromosomes in *Psathyrostachys juncea* and most *Leymus* species containing the Ns genome. Furthermore, six wheat*–P. huashanica* chromosome addition lines (i.e., 2Ns, 3Ns, 4Ns, 7Ns chromosomes and chromosomal segments) were characterized using the newly developed FISH markers. Thus, these probes can rapidly and precisely detect *P. huashanica* alien chromosomes in the wheat background.

**Conclusions:**

The FISH karyotype established in this study lays a solid foundation for the efficient identification of *P. huashanica* chromosomes in wheat genetic improvement programs.

## Background

*Psathyrostachys huashanica* Keng (2n = 2x = 14, NsNs), a diploid, perennial, cross-pollinating species belonging to the genus *Psathyrostachys* Nevski in the tribe Triticeae, is distributed primarily in the Huashan Mountain region of Shanxi province, China [1]. There has been considerable interest in *P. huashanica* among wheat breeders because it is a promising wild relative of common wheat [2]. It has many agronomically valuable characteristics, including early maturation, the production of multiple kernels and tillers, drought tolerance, and resistance to biotic factors (e.g., pathogens causing stripe rust, leaf rust, take-all, and powdery mildew) [3]. Moreover, it has been used as a tertiary gene resource for wheat genetic improvement ever since chromosome-mediated gene transfers from *P. huashanica* into common wheat were initiated in the 1990s [2]. Progeny lines carrying *P. huashanica* chromosomes or chromosomal segments incorporated into the wheat genome were subsequently developed as lines with chromosomal additions [4–6], substitutions [7, 8], and translocations [9, 10]. These lines outperformed their wheat parents in terms of stress resistance and agronomic traits, making them valuable germplasm for wheat breeding. Molecular markers, including random-amplified polymorphic DNA markers targeting the *P. huashanica* Ns genome [11] and sequence characterized amplified region markers specific for the 1Ns, 2Ns, 3Ns, and 5Ns chromosomes [4, 12–14], have been developed for exogenous chromatin tracking.

Fluorescence *in situ* hybridization (FISH) is a widely used cytogenetic tool for detecting chromosomal aberrations as well as for studying chromosomal behavior and the genomic localization of repetitive DNA sequences [15, 16]. The FISH karyotype established on the basis of informative probe labeling patterns on chromosomes may provide researchers with landmarks useful for identifying chromosomes. In previous FISH studies, repetitive sequence probes, such as pSc119.2, pTa535, pTa713, pAs1, and microsatellites like (AAC)_5_, enabled the convenient identification of wheat chromosomes according to distinct probe hybridization patterns [17–20]. Such FISH karyotye has also been conducted in investigations of the diploid *Thinopyrun elongatum* [21], *Dasypyrum villosum* [22], *Agropyron cristatum* [23], *Secale cereale* [24], *Hordeum vulgare* [25], and *Aegilops* species, including *Ae. markgrafii*, *Ae. cylindrica*, *Ae. triuncialis* [26], *Ae. comosa*, *Ae. geniculata*, *Ae. biuncialis* and *Ae. umbellulata* [27, 28]. To date, few repetitive sequences have been used as FISH probes for analyzing *P. huashanica* chromosomes. Furthermore, a detailed FISH karyotype based on homoeologous relationships with wheat is not available for the *P. huashanica* Ns-genome chromosomes. A lack of a standard *P. huashanica* karyotype has greatly hindered the chromosome-mediated transfer of elite genes from *P. huashanica* into cultivated wheat.

Although FISH using repetitive sequence probes has been widely used for identifying chromosomes in numerous Triticeae species [19–29], it is not a viable option for determining inter-genera chromosomal homoeologous relationships because of the broad variation in these repetitive elements among diverse genomes [30, 31]. Chromosome painting using chromosome-specific probes, which are designed on the basis of single-copy sequences, is a novel cytological method for diagnosing chromosomal abnormalities, detecting chromosomal rearrangements, investigating chromatin organization, and constructing ancestral karyotypes [32]. Li et al. [33] recently developed seven oligonucleotide pools (Synt1-Synt7) derived from the conserved sequences in the collinear chromosomal regions of each linkage group in wheat and barley. These pools were used to differentiate between the chromosomal homeologous groups in the genera *Secale*, *Aegilops*, *Thinopyrum*, and *Dasypyrum*. Accordingly, these newly synthesized linkage group-specific bulked oligonucleotide pools are applicable for investigating chromosomal homoeologous relationships among Triticeae species.

In our previous studies, we generated hybrids from a cross between *P. huashanica* and common wheat without an embryo rescue step and obtained hybrid derivatives by backcrossing and selfing [34]. A detailed FISH karyotype of *P. huashanica* chromosomes remains unavailable and there are relatively few *P. huashanica*-specific molecular markers. This has made it difficult to identify wheat–*P. huashanica* progenies useful for breeding. To optimize the utility of *P. huashanica* desirable genes for the genetic improvement of wheat via chromosome engineering, FISH-based markers that can efficiently identify *P. huashanica* chromosomes in the wheat background are urgently needed. Thus, the objectives of this study were to: (1) develop a FISH-based karyotype specific for the *P. huashanica* Ns-genome chromosomes; (2) validate the *P. huashanica* chromosome-specific FISH markers in common wheat and wheat-related species; and (3) identify wheat–*P. huashanica* derivative lines using the developed FISH markers.

## Materials and methods

### Plant materials

Specific details regarding the plant materials used in this study are provided in Tables 1 and 2. Randomly selected individual plants from two *P. huashanica* accessions (2n = 2x = 14, NsNs) were used to generate a FISH karyotype. The *P. huashanica* chromosome-specific FISH markers were validated using the following species: common wheat landrace Chinese Spring (CS; 2n = 6x = 42, AABBDD); the wheat-related species *Secale cereale* (2n = 2x = 14, RR), *Hordeum vulgare* (2n = 2x = 14, II), *Thinopyrum elongatum* (2n = 2x = 14, EE), *Agropyron cristatum* (2n = 2x = 14, PP), *Dasypyrum villosum* (2n = 2x = 14, VV), *Pseudoroegneria stipifolia* (2n = 2x = 14, StSt), *Psathyrostachys juncea* (2n = 2x = 14, NsNs), *Leymus secalinus* (2n = 4x = 28, NsNsXmXm), *L. pseudoracemosus* (2n = 4x = 28, NsNsXmXm), *L. multicaulis* (2n = 4x = 28, NsNsXmXm), *L. racemosus* (2n = 4x = 28, NsNsXmXm), *L. coreanus* (2n = 4x = 28, NsNsXmXm), and *L. arenarius* (2n = 8x = 56, Ns_1_Ns_1_Ns_2_Ns_2_XmXmXmXm). Six wheat–*P. huashanica* derivatives (18-2-6, 19-2-5, 20-1-1, 23-1-1, 26-3-2, and 32-2-5) were obtained via the hybridization between CS*ph2b* and *P. huashanica* accession ZY3157. Designation of genome formulas were according to Yen and Yang [35–37]. Regarding the genomic *in situ* hybridization (GISH) analysis, the CS genome was used as the blocking DNA, whereas the *P. huashanica* genome was used as the source of the probe DNA. CS and *Secale cereale* QL were maintained in our laboratory. Materials with PI numbers were kindly provided by The U.S. National Plant Germplasm System (NPGS), and materials with ZY numbers were collected by Yen. Chi and J. L. Yang (Triticeae Research Institute, Sichuan Agricultural University). Voucher specimens have been deposited in the herbarium of the Triticeae Research Institute, Sichuan Agricultural University, China.

**Table 1:** Plant materials used in this study

Materials	Accession	Origin	Ploidy	Genome
Chinese Spring	CS	China	6×	ABD
*Psathyrostachys huashanica*	ZY3156	China	2×	Ns
*Psathyrostachys huashanica*	ZY3157	China	2×	Ns
*Psathyrostachys juncea*	PI314082	Former, Soviet Union	2×	Ns
*Secale cereale*	QL	China	2×	R
*Hordeum vulgare*	ZY11001	China	2×	I
*Agropyron cristatum*	PI499389	China	2×	P
*Dasypyrum villosum*	PI470279	Turkey	2×	V
*Thinopyrum elongatum*	PI531718	Tunisia	2×	E
*Pseudoroegneria stipifolia*	PI325181	Russian	2×	St
*Leymus pseudoracemosus*	PI531810	China	2×	NsXm
*Leymus racemosus*	ZY07023	China	4×	NsXm
*Leymus secalinus*	ZY09002	China	4×	NsXm
*Leymus coreanus*	PI531578	Russian	4×	NsXm
*Leymus multicaulis*	PI440326	Kazakhstan	4×	NsXm
*Leymus arenarius*	PI294582	Sweden	8×	NsXm

**Table 2:** Pedigree and chromosome constitution of six hybrid lines used in this study

Line	Chromosome constitution	Pedigree
18-2-6	2n = 42 W+II7Ns	(CS*ph2b*×*P. huashanica*)×CS×CS-F_4_
19-2-5	2n = 42 W+I2Ns+ItNs	(CS*ph2b*×*P. huashanica*)×CS×CS-F_4_
20-1-1	2n = 42 W+IIt2NsL	(CS*ph2b*×*P. huashanica*)×CS×CS-F_4_
23-1-1	2n = 42 W+IItNs	(CS*ph2b*×*P. huashanica*)×CS-F_4_
26-3-2	2n = 42 W+I2Ns+I4Ns	(CS*ph2b*×*P. huashanica*)×CS-F_4_
32-2-5	2n = 42 W+I3Ns	(CS*ph2b*×*P. huashanica*)×CS-F_5_

Ns: Ns-genome chromosomes of *P. huashanica*, W: A-, B- and D-genome chromosomes of wheat

### **Development of*P. huashanica*chromosome-specific probes**

Actively growing root tips from germinating seeds or growing plants were cut and treated with nitrous oxide for 2.5 h and 70% (v/v) acetic acid for 5 min before being digested with pectinase and cellulase (Yakult Pharmaceutical Industry Co., Ltd., Tokyo, Japan) [19]. Root tips for outcrossing species were taken from randomly selected individual plants or seed of each accession. The FISH and GISH slides were prepared as described by Han et al. [38]. To construct the *P. huashanica* karyotype, the pSc200, pTa71A-2, and Oligo-44 repetitive sequences were selected for the FISH experiments after screening other oligonucleotides probes, including pSc119.2, pTa535, pTa713, pAs1, (CTT)_12_, and (AAC)_5_. Sequence of each probe was listed in Table 3 and all probes were 5’ end-labelled with 6-carboxyfluorescein or 6-carboxytetramethylrhodamine by Sangon (Shanghai, China). The hybridization was completed according to a slightly modified version of the method described by Han et al. [38]. Briefly, 10 µL hybridization solution comprising the probes and buffer [0.5 µL each probe in 2× saline sodium citrate (SSC) and 1× TE buffer] was added to each slide. The samples were denatured at 80 °C for 5 min and then incubated at 37 °C for 2 h, after which they were washed with 2× SSC buffer. The chromosomes were counterstained with a DAPI (4′,6-diamidino-2-phenylindole) solution (Vector Laboratories, Inc., Burlingame, CA, USA) before photomicrographs of the chromosomes were taken using the DP80 CCD camera (Olympus, Tokyo, Japan) installed on the BX-63 microscope (Olympus). The photomicrographs were processed using Photoshop CS5.0 (Adobe Systems Incorporated, San Jose, CA, USA).

**Table 3:** Probes used in this study

Probe	Sequences (5′-3′)	Reference
pSc119.2	CCGTTTTGTGGACTATTACTCACCGCTTTGGGGTCCCATAGCTAT	[17]
pTa535	AAAAACTTGACGCACGTCACGTACAAATTGGACAAACTCTTTCGGAGTATCAGGGTTTC	[17]
pAs1	CCTTTCTGACTTCATTTGTTATTTTTCATGCATTTACTAATTATTTTGAGCTATAAGAC	[17]
(AAC)_5_	(AAC)_5_	[20]
pTa713	AGACGAGCACGTGACACCATTCCCACCCTGTCTTAGCG	[39]
(CTT)_12_	(CTT)_12_	[40]
pTa71A-2	CCGACGGCCGTCGTGGACGGAAGTTGACGCGCGCCATGGAAAACTG	[41]
Oliog-44	TAGCTCTACAAGCTAGTTCAAATAATTTTACACTAGAGTTGAAC	[42]
pSc200	CTCACTTGCTTTGAGAGTCTCGATCAATTCGGACTCTAGGTTGATTTTTGTATTTTCT	[43]
Synt1-Synt7	\	[33]

Probes Synt1-Synt7 were kindly provided by Prof. Z.J. Yang (Center for Informational Biology, School of Life Science and Technology, University of Electronic and Technology of China)

### **Karyotyping of*P. huashanica*chromosomes**

To distinguish the *P. huashanica* homoeologous chromosomes from the wheat chromosomes, bulked oligonucleotide libraries (Synt1-Synt7) kindly provided by Prof. Z.J. Yang (Center for Informational Biology, School of Life Science and Technology, University of Electronic and Technology of China) were used for FISH painting. The washed slides were used for a FISH involving the oligonucleotide probes pTa71A-2 and Oligo-44. The FISH painting was performed as described by Han et al. [44] and Bi et al. [45]. Slides were washed as described by Komuro et al. [19]. Photomicrographs were taken as described in the FISH protocol. Fifteen well-spread metaphase cells of *P. huashanica* accession ZY3157 were selected for an analysis of chromosomal parameters. The relative length was calculated by dividing the length of a particular chromosome by the total length of chromosomes in the haploid set. The chromosomal arm ratio was determined by dividing the length of the longer arms by the length of the shorter arms. Data were processed using the Image J software and Microsoft Office Excel 2010.

### **Validation of the*P. huashanica*-specific FISH-based markers**

To validate the specificity and utility of the developed cytological markers, pSc200, pTa71A-2, and Oligo-44 were used for a FISH analysis of CS, 13 wheat relatives, and wheat–*P. huashanica* derivatives. A GISH was performed using the washed FISH slides for the derived lines to confirm the presence of *P. huashanica* chromatin. For both *P. huashanica* and CS, genomic DNA was extracted from fresh leaves according to the cetyltrimethylammonium bromide method [46]. The *P. huashanica* (ZY3157) genomic DNA was labeled with Texas Red-12-dUTP in the nick translation mix (Thermo Fisher Scientific, Eugene, OR, USA) and then used as the GISH probe. The CS DNA was used for blocking. The GISH was performed according to a modified version of the method described by Han et al. [38]. Specifically, 10 µL GISH hybridization solution containing 100 ng labeled probe DNA and blocking DNA (1:150 probe DNA:blocking DNA ratio), 2× SSC, 10% (v/v) dextran sulfate, and 50% (v/v) formamide was added to each slide. The samples were denatured at 85 °C for 5 min, incubated at 50 °C overnight, and washed with 2× SSC buffer. The chromosomes were counterstained and photomicrographs were taken as described in the FISH protocol.

## Results

### Karyotyping of ***P. huashanica*** Ns-genome chromosomes

**Fig. 1:**
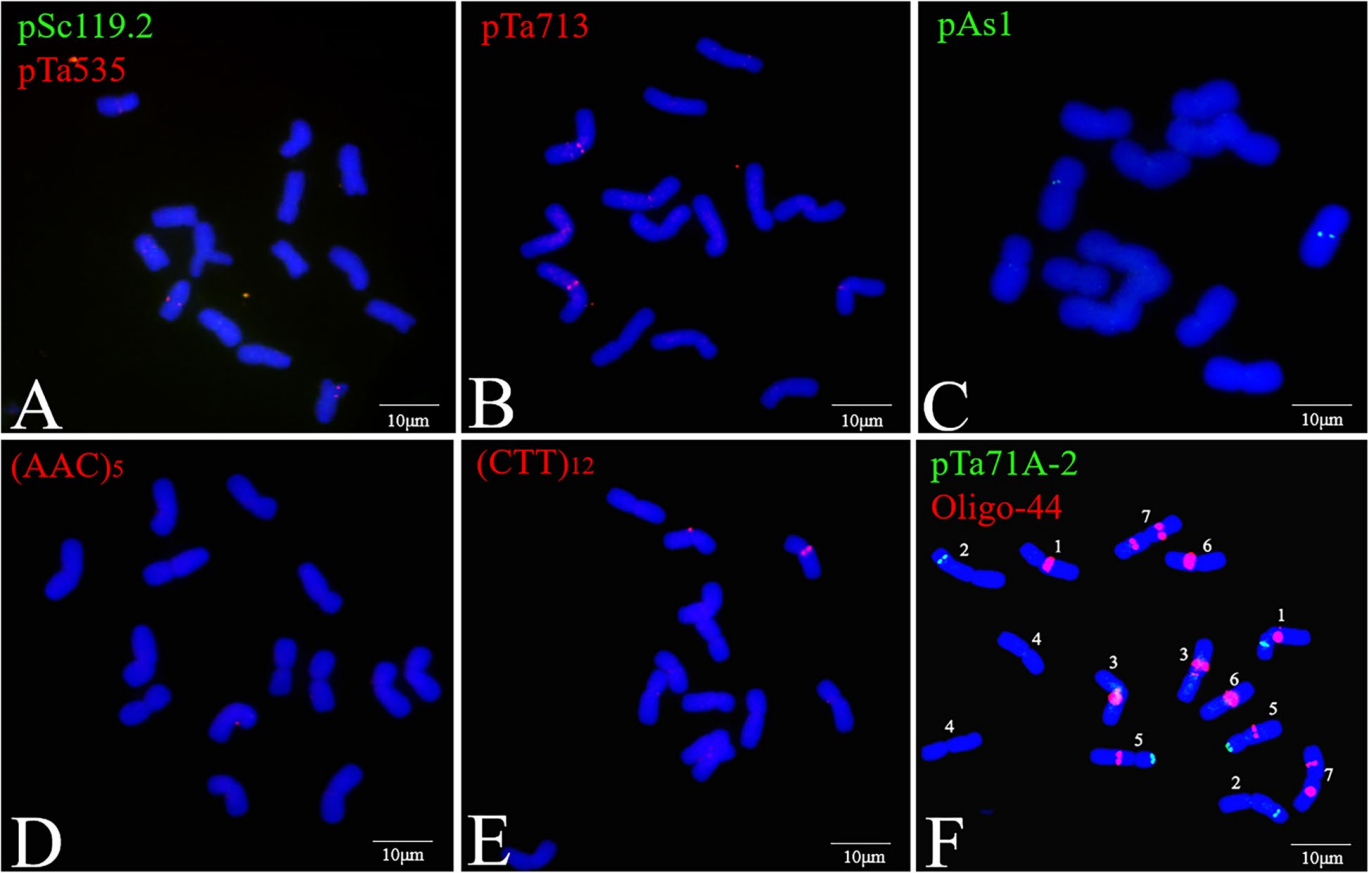
FISH with oligonucleotide probes on chromosomes of *P. huashanica* accession ZY3156. **A-F** FISH patterns of probes pSc119.2 (green) + pTa535 (red), pTa713 (red), pAs1 (green), (AAC)_5_ (red), (CTT)_12_ (red) and pTa71A-2 (green) + Oligo-44 (red). Scale bar: 10 μm

**Fig. 2:**
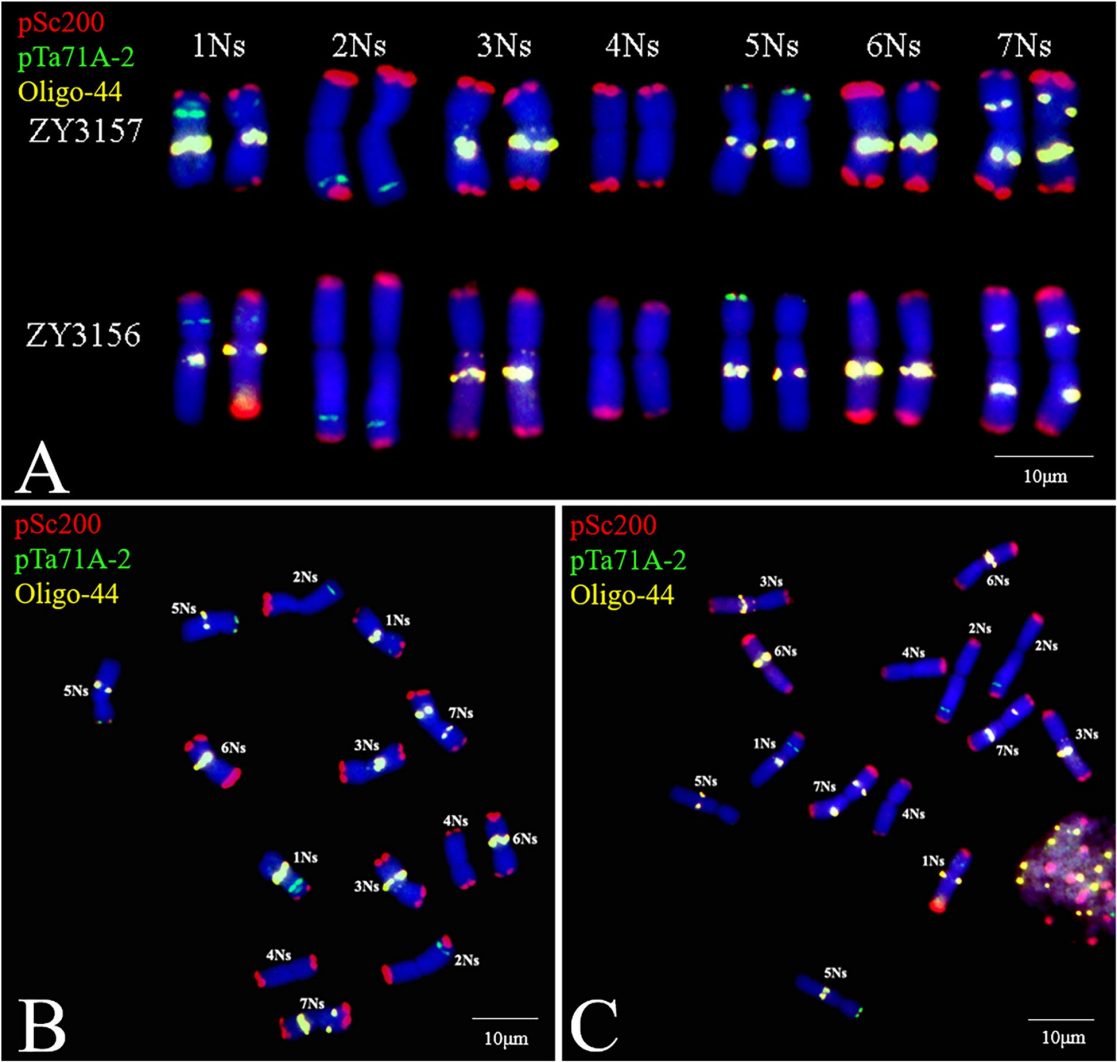
FISH with oligonucleotide probes on chromosomes of *P. huashanica* accession ZY3156 and ZY3157. **A** FISH patterns of 1-7Ns chromosomes using probes pSc200 (red), pTa71A-2 (green) and Oligo-44 (yellow). **B**, **C** FISH identification in accessions ZY3157 and ZY3156 by probes pSc200 (red), pTa71A-2 (green) and Oligo-44 (yellow). Scale bar: 10 μm

**Fig. 3:**
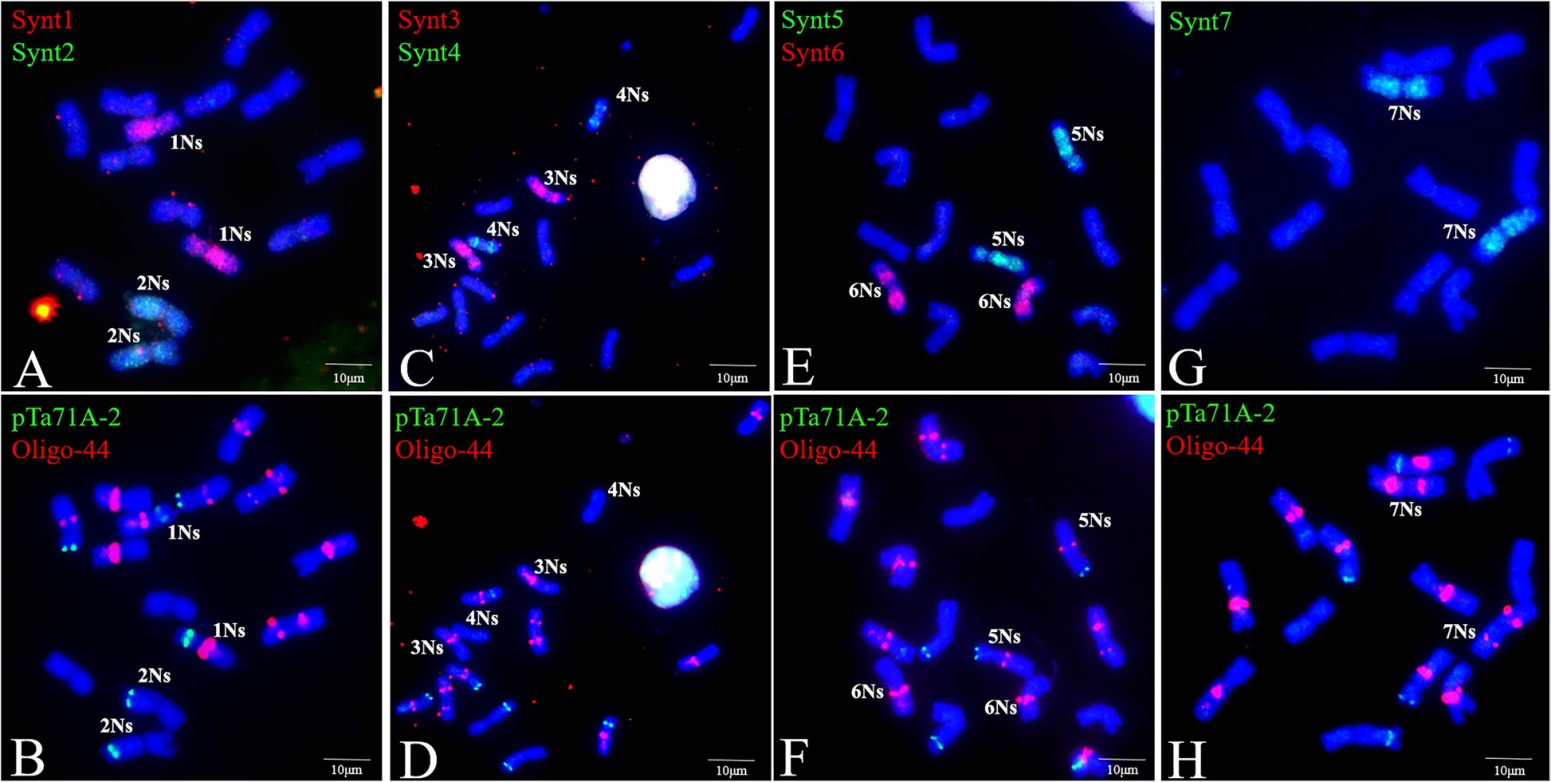
FISH painting using bulked oligo probes and sequential FISH with oligonucleotide probes on chromosomes of *P. huashanica* accession ZY3157. (**A**, **B**, **C**, **D**, **E**, **F**, **G**, **H**) FISH painting using bulked oligo probes Synt1 (red) + Synt2 (green), Synt3 (red) + Synt4 (green), Synt5 (green) + Synt6 (red) and Synt7 (green) with corresponding FISH using probes pTa71A-2(green) + Oligo-44(red). Scale bar: 10 μm

Several DNA probes, including pSc119.2, pTa535, pTa713, pAs1, (AAC)_5_, (CTT)_12_, pSc200, pTa71A-2, and Oligo-44, were used for the FISH analysis of *P. huashanica* chromosomes (Figs. 1 and 2B C). The FISH results indicated that the pTa71A-2 and Oligo-44 probe combination unambiguously discriminated the *P. huashanica* chromosomes (Fig. 1 F). The probes pTa71A-2 and Oligo-44, which were associated with pSc200, enabled the precise identification of all *P. huashanica* Ns-genome chromosomes (Fig. 2B C). By combining the oligonucleotide probes for FISH images and the bulked pool probes (Synt1–Synt7) for the seven Triticeae linkage groups (Fig. 3), the FISH patterns of *P. huashanica* chromosomes 1Ns to 7Ns were established (Fig. 2 A). Details regarding chromosome morphology are summarized in Table 4.

**Table 4:** Chromosome parameters of *P. huashanica*

Chromosome	Arm ratio	Relative length (%)
1Ns	1.60 ± 0.15	13.42 ± 0.87
2Ns	1.34 ± 0.11	16.37 ± 0.85
3Ns	1.48 ± 0.12	14.73 ± 0.71
4Ns	1.28 ± 0.08	12.39 ± 0.70
5Ns	2.02 ± 0.17	13.68 ± 0.53
6Ns	1.10 ± 0.06	13.37 ± 0.95
7Ns	1.07 ± 0.05	16.07 ± 0.72

The data represent the mean (± standard deviation) of fifty cells

The chromosomes of the two *P. huashanica* accessions had similar probe hybridization patterns, with some variations in the FISH signals for the 1Ns homologous chromosomes analyzed using probes pTa71A-2 and pSc200, and for 2Ns analyzed using probe pSc200, which hybridizes exclusively to the terminal region of all chromosomes (Fig. 2 A). Chromosome 1Ns was characterized by heteromorphic pTa71A-2 signals, with varying densities at the nucleolar organizing regions (NORs) of the short arms among the homologous chromosomes. Additionally, strong Oligo-44 signals were detected near the centromere region of the long arms. Terminal pSc200 signals were observed at the short arms of chromosome 1Ns. Moreover, pSc200 revealed the heteromorphism at the terminal region of the long arms. Chromosome 2Ns had clear pTa71A-2 signals at the subterminal region of the long arms. The variation at the terminal region of the 2Ns chromosomes detected using pSc200 was identical to that on the 1Ns chromosomes. Chromosome 3Ns was identified on the basis of weak Oligo-44 signals in pericentromeric and strong Oligo-44 signals near the centromere region of the long arms; strong terminal pSc200 signals were detected on both arms. Chromosome 4Ns lacked specific pTa71A-2 and Oligo-44 signals, but strong terminal pSc200 signals were observed on both arms. Clear pTa71A-2 and faint pSc200 signals were simultaneously produced at the terminal of the short arms of 5Ns chromosomes, with strong Oligo-44 signals at the intercalary region of the long arms. Chromosome 6Ns had strong Oligo-44 signals near the centromere region of the long arms and clear terminal pSc200 signals on both arms. Notably, distinct Oligo-44 signals were observed at the middle region of both arms of chromosome 7Ns. Terminal pSc200 signals on chromosome 7Ns were the same as those on chromosomes 3Ns, 4Ns, and 6Ns (Fig. 2 A).

### Validation of the FISH markers

**Fig. 4:**
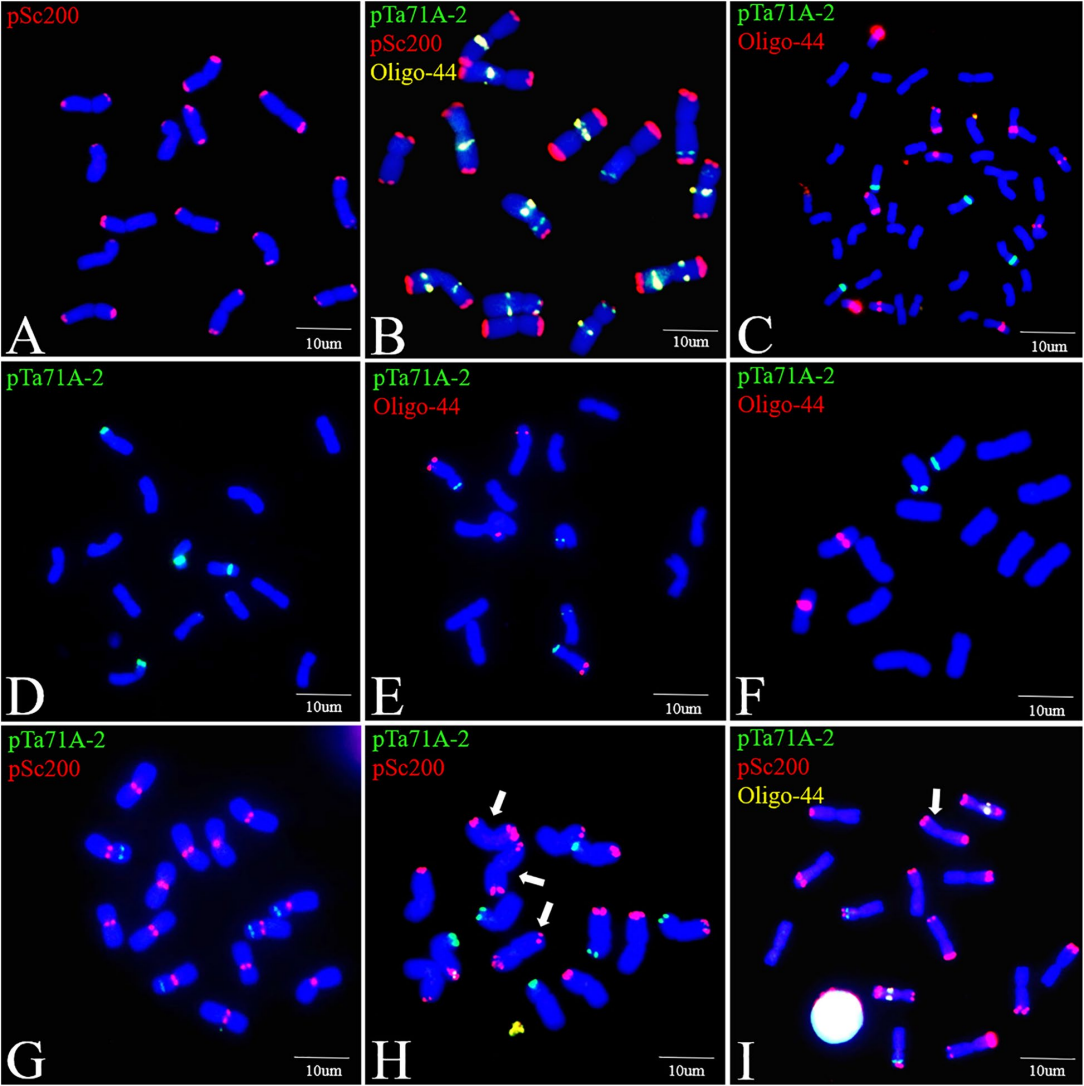
FISH with oligonucleotide probes on chromosomes of *P. huashanica* accession ZY3157, common wheat Chinese Spring (CS) and diploid wheat relatives. **A-G** FISH patterns of probes pSc200, pTa71A-2 or Oligo-44 on chromosomes of **A**, **B** *P. huashanica*, **C** CS, **D** *Pse. stipifolia*, **E** *Th. elongatum*, **F** *Das. villosum*, **G** *H. vulgare*, **H** *A. cristatum* and **I** *S. cereale*, respectively. White arrows indicate the chromosomes which can’t be discriminated from the 4Ns chromosomes of *P. huashanica* using all the three probes. Scale bar: 10 μm

To validate the specificity of the FISH markers for *P. huashanica* Ns-genome chromosomes, probes pTa71A-2, Oligo-44, and pSc200 were used to perform a FISH analysis of the metaphase cells of CS and six diploid wheat relatives containing St, E, V, I, P and R genomes, respectively (Table 1). There was a lack of pSc200 signals on all the chromosomes of wheat, *Pse. stipifolia*, *Th. elongatum*, and *Das. villosum*. However, pSc200 signals were detected on the *H. vulgare* I-genome chromosomes, but only at the centromeric regions. Therefore, a FISH involving pSc200 can distinguish the *P. huashanica* Ns-genome chromosomes from the A-, B-, D-, St-, E-, V-, and I-genome chromosomes. Oligo-44 generated signals with varying intensities on a few chromosomes of CS, *Th. elongatum*, *Das. villosum*, and *S. cereale*, whereas pTa71A-2 revealed polymorphic hybridization sites on several chromosomes in all species. Combining pSc200 with Oligo-44 or pTa71A-2 enabled the discrimination of all *P. huashanica* Ns-genome chromosomes, with the exception of the 4Ns chromosomes, from the P- and R-genome chromosomes, some of which had terminal pSc200 signals that were the same as those on the 4Ns chromosomes (Fig. 4).

**Fig. 5:**
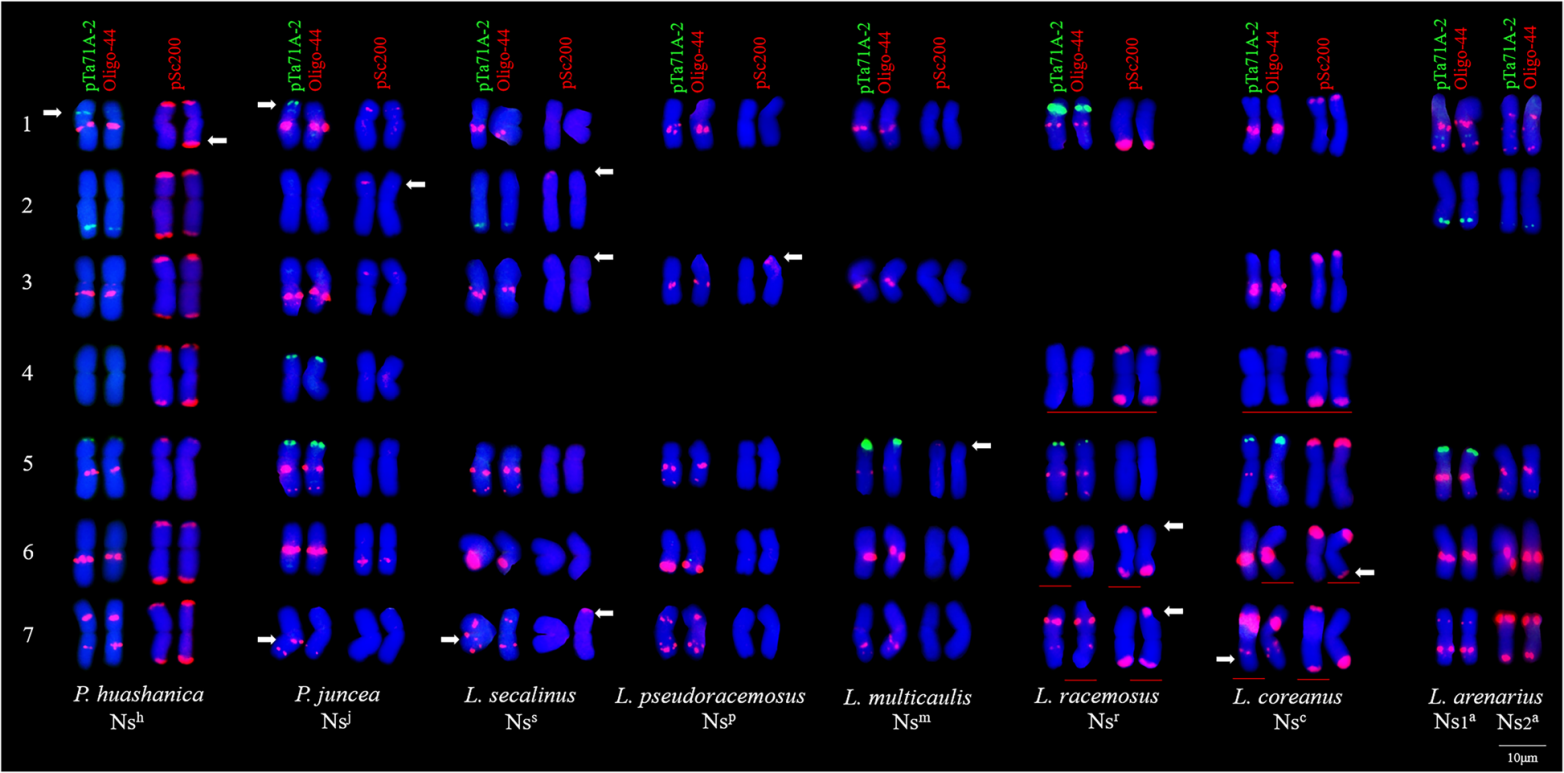
Chromosome FISH karyotype of the Ns-genome chromosomes using probes pSc200 (red), pTa71A-2 (green) and Oligo-44 (red) for *P. juncea* and *Leymus* species. The arrangement of chromosomes was based on the FISH patterns similarities with *P. huashanica* (ZY3156) chromosomes and chromosomes morphology including arm ratio and relative length. White arrows indicate the FISH signal variations within homologous chromosomes, red lines indicate the chromosomes which can’t be discriminated from chromosomes of *P. huashanica* using all the three probes. Scale bar: 10 μm

These probes were also used for a FISH analysis of seven Ns-genome-containing species to assess their utility for distinguishing between *P. huashanica* chromosomes and other Ns-genome chromosomes in a different genetic background. The pSc200 signals were mainly detected at the terminal region of one or both arms of the Ns-genome chromosomes in most of the examined species. The exceptions were *P. juncea*, in which faint signals were detected primarily at the subterminal region of several chromosomes, and *L. arenarius*, in which signals were undetectable. The pSc200 signal distribution patterns were not specific to particular chromosomes or genome types, but the strong terminal signals on all *P. huashanica* chromosomes except chromosome 5Ns were useful for distinguishing from the Ns^j^, Ns^s^, Ns^p^, Ns^m^, and Ns^a^ chromosomes to some extent. All of the identified Ns-genome chromosomes in the different species had Oligo-44 FISH patterns that were similar to those of *P. huashanica* chromosomes belonging to homologous groups 1, 3, 5, 6, and 7, but there were obvious interspecific differences in the signal localization for the 1Ns_1_^a^ and 1Ns_2_^a^ chromosomes, all 5Ns chromosomes (except for 5Ns^m^ and 5Ns^c^), chromosome pairs 6Ns^s^, 6Ns^p^, 7Ns^j^, 7Ns^p^, 7Ns^m^, 7Ns_2_^a^, and a single 7Ns^s^ and 7Ns^c^ chromosome. The mapping of the pTa71A-2 sites on these Ns-genome chromosomes revealed different 45 S rDNA loci that were the same or similar in size, which provided limited information useful for discriminating between chromosomes (Fig. 5).

Using the three probes simultaneously for a FISH analysis unambiguously distinguished chromosome 1Ns^h^ from the homologous chromosomes in all other species. Chromosome 2Ns^h^ could also be distinguished from all other 2Ns chromosomes by the pSc200 and pTa71A-2 probe combination. The pSc200 and Oligo-44 probes were able to differentiate chromosome 3Ns^h^ from the other 3Ns chromosomes. Probes pTa71A-2 and Oligo-44 failed to hybridize to chromosome 4Ns^h^, whereas pSc200 was able to discriminate this chromosome from chromosomes 4Ns^j^, 4Ns^s^, 4Ns^p^, 4Ns^m^, and 4Ns^a^. The pSc200, Oligo-44, and pTa71A-2 probe combination helped separate chromosome 5Ns^h^ from all other 5Ns chromosomes. The Ns^h^ and other Ns chromosomes (except for Ns^r^ and Ns^c^) in homologous groups 6 and 7 could also be clearly distinguished using the pSc200 and Oligo-44 probe combination (Fig. 5).

### **Utility of the FISH markers for identifying*P. huashanica*chromosomes in the wheat background**

**Fig. 6:**
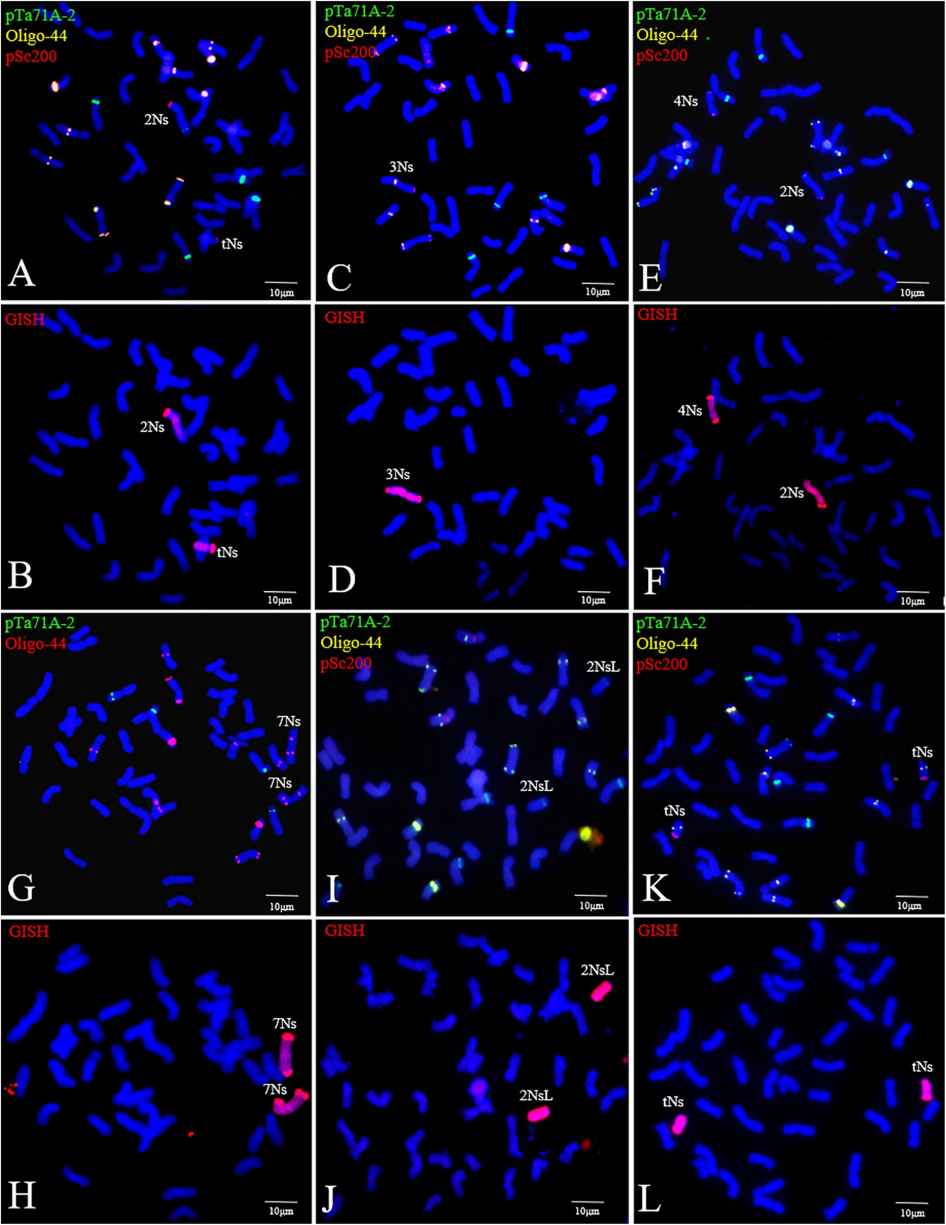
Chromosome identification of the wheat-*P. huashanica* chromosomal addition lines. Images for FISH results using probes pTa71A-2 (green), pSc200 (red) and/or Oligo-44(red/yellow), and the sequential GISH (red) of corresponding spreads. **A**, **B** Line 19-2-5 had additional 2Ns and tNs chromosomes. **C**, **D** Line 32-2-5 had one 3Ns chromosome. **E**, **F** Line 26-3-2 had additional 2Ns and 4Ns chromosome. **G**, **H** Line 18-2-6 had a pair of 7Ns chromosomes. **I**, **J** Line 20-1-1 had two t2NsL chromosomes. **K**, **L** Line 23-1-1 had additional tNs chromosomes. Scale bar: 10 μm

To evaluate the potential utility of pTa71A-2, Oligo-44, and pSc200 for identifying *P. huashanica* chromosomes in the wheat background, a FISH was performed on the metaphase cells of wheat–*P. huashanica* hybrid derivatives. Six wheat–*P*. *huashanica* chromosome addition lines with diverse chromosomal contents were unambiguously characterized. The FISH results indicated that line 19-2-5 (2n = 44) was a chromosome addition line containing a 2Ns and telosomic chromosome (Fig. 6 A, 6B). Line 32-2-5 (2n = 43) was identified as a monosomic chromosome addition line containing a 3Ns chromosome on the basis of the signal patterns and a larger arm ratio than that of the 6Ns chromosomes (Fig. 6C, D). The probe signals indicated that line 26-3-2 (2n = 44) carried an additional 2Ns and 4Ns chromosome respectively (Fig. 6E F), whereas line 18-2-6 (2n = 44) was a disomic chromosome addition line with a pair of 7Ns chromosomes (Fig. 6G H). Line 20-1-1 (2n = 44) was a 2NsL ditelosomic addition line. Although line 23-1-1 (2n = 44) was also identified as a ditelosomic addition line, it was unclear which group the additional chromosomes belonged to (Fig. 6I L).

## Discussion

The ability to accurately identify alien chromosomes in the wheat background is critical for exploiting alien genetic resources [39]. Accordingly, FISH analyses involving pSc119.2, pTa535, pTa713, pTa794, pAs1, and the microsatellites (AAC)_5_ and (CTT)_12_ have been widely conducted to identify chromosomes from wheat [17], *S. cereale* [47], diploid *Th. elongatum* [21], *Das. villosum* [22], and *Aegilops* species (S-, U-, C-, N-, D-and M-genomes) [26–28, 32, 48, 49]. However, the resolution of the results obtained by combining these frequently used FISH probes was insufficient for discriminating the seven pairs of *P. huashanica* chromosomes (Fig. 1). By conducting a FISH analysis using repetitive sequences, we revealed that all *P. huashanica* Ns-genome chromosomes can be unambiguously discriminated from each other by pTa71A-2 and Oligo-44 (Fig. 1 F). Moreover, the detailed FISH karyotype constructed using pSc200, pTa71A-2, and Oligo-44 enabled the accurate identification of all *P. huashanica* chromosomes (Fig. 2). Badaeva et al. [50] developed the FISH karyotypes of 12 diploid *Aegilops* species and divided the chromosomes into homoeologous groups according to the standard C-banding patterns. Zhang et al. [51] constructed a FISH-based map of *H. villosa* chromosomes on the basis of the FISH patterns on the V-genome chromosomes from a whole set of wheat*–H. villosa* disomic chromosome addition lines. Said et al. [23] arranged the P-genome chromosomes by mapping conserved single-copy wheat cDNAs on the homoeologous chromosomes of *A. cristatum*. Using linkage group-specific bulked oligonucleotide pools, we constructed the *P. huashanica* karyotype relatively simply and quickly. Notably, FISH using pSc200, Oligo-44, and pTa71A-2 together distinguished the *P. huashanica* Ns-genome chromosomes from the wheat chromosomes as well as all chromosomes except for 4Ns from the chromosomes of diploid wheat relatives containing St, E V, I, P and R genomes, while also differentiating between the *P. huashanica* Ns-genome chromosomes in all homologous groups and those of *P. juncea* and most *Leymus* species.

A previous study proved that for blotting and FISH analyses, pSc200 from rye telomeric/subtelomeric repetitive sequences does not produce detectable signals in the wheat genome [52]. Hence, it was used to differentiate the R-genome chromosomes of rye from the A-, B-, and D-genome chromosomes of wheat [43]. It has also been used to detect changes in the telomeric/subtelomeric sequences of rye chromosomes in wheat–rye amphidiploids [53]. In this study, the terminal pSc200 signals on all *P. huashanica* chromosomes enabled the discrimination of the *P. huashanica* chromosomes from the wheat chromosomes and revealed the variation of telomeric sequences from the Ns-genome chromosomes in *P. juncea* and *Leymus* species (Figs. 2 and 4 C, and 5). Probe pTa71A-2, which belongs to the pTa71 family of repeating sequences (45 S rDNA), comprises a 9-kb sequence from the common wheat 18 S–5.8 S–26 S rDNA and intergenic spacers [41, 54]. The 45 S rDNA gene locus is usually linked with NORs in the tribe Triticeae. Previous research indicated that major NORs are preferentially located on the short arms of chromosomes in homoeologous groups 1, 5, and 6 [54, 55]. However, according to the results of this study, pTa71A-2 hybridizes to the short arms of the 1Ns and 5Ns chromosomes while the long arms of the 2Ns chromosomes (Fig. 2 A). The positions of major NORs determined using pTa71A-2 in this study were in accordance with the findings of an earlier study by Kisimi [56]. Thus, the differences in the major 45 S rDNA sites on the long arms of the 2Ns chromosomes in *P. huashanica* were likely due to a chromosomal non-homologous recombination between 6Ns and 2Ns. Li et al. [33] confirmed the non-homologous chromosomal rearrangements in rye, *Ae. uniaristata*, *Ae. markgrafii*, and *Ae. umbellulata* using linkage group-specific bulked oligo pools. In contrast, we did not detect interchanges involving *P. huashanica* 2Ns and 6Ns chromosomes because the probe pools mainly hybridized to the non-terminal region of both arms of the *P. huashanica* chromosomes (Fig. 3 A, C, E, and G), indicative of a relatively low homology between the telomeric regions of wheat and *P. huashanica* chromosomes. Oligo-44 is a new tandem repeat isolated from the CS genome that has been used to identify specific wheat chromosomal segments [42]. In the current study, abundant Oligo-44 hybridization signals facilitated the identification of *P. huashanica* chromosomes, and they also helped the detection of several Ns-genome chromosomes in *P. juncea* and *Leymus* species (Fig. 5), which contain the Ns genome from the genus *Psathyrostachys* and the Xm genome from unconfirmed donors [57]. In addition to the pSc200 and pTa71A-2 signals, polymorphic Oligo-44 hybridization patterns elucidated the genetic differences among the Ns-genome chromosomes in diverse genetic backgrounds (Fig. 5), which were previously determined in Southern blots and via molecular studies [58–60].

The distribution of Oligo-44 signals provided some insights into the relationship between Ns and Xm genomes. On the basis of the chromosome pairing in intergeneric hybrids and the results of DNA hybridization experiments, the evolutionary distance between the Ns and Xm genomes in *Leymus* species has been investigated. In earlier studies by Dewey [61] and Wang et al. [62], the trivalent ratio was low during analyses of the configuration of F_1_ pollen mother cells at metaphase I in *Leymus* × *Psathyrostachys* hybrids, suggesting the tetraploid *Leymus* species are allotetraploids. Zhang et al. [63] subsequently confirmed their findings using GISH and chromosome pairing data. However, FISH analyses involving the mapping of dispersed retrotransposon-like repeats from *L. mollis* and *L. arenarius* on the chromosomes of *Psathyrostachys* and *Leymus* species indicated that *Leymus* species should be considered as autopolyploids with the Ns genome or segmental allopolyploids consisting of an altered basic Ns genome [64]. The Oligo-44 signal distribution characteristics among the Ns-genome chromosomes indicated that the corresponding repetitive sequence was conserved during the evolution of the Ns genome (Fig. 5; Table 5). Thus, there are theoretically 2-times and 4-times more chromosomes carrying Oligo-44 signals of assumed autotetraploid and autooctaploid *Leymus* species, respectively, than in diploid Ns-genome-containing species. In the current study, the number of chromosomes giving Oligo-44 hybridization sites was 10–13 in tetraploid *Leymus* species and 22 in the octaploid *L. arenarius* (Table 5), implying that *Leymus* species are likely not autopolyploids, which is consistent with the available DNA sequence information [65, 66]. Because small-scale analyses may lead to incomplete conclusions, more comprehensive approaches are required for future biosystematics-based investigations of the genus *Leymus*.

**Table 5:** Chromosome numbers with FISH signals of each probe observed most for the Ns-genome- containing species

Species	Ploidy	Oligo-44	pSc200	pTa71A-2
*P. huashanica*	2×	10	14	6
*P. juncea*	2×	10	7	6
*L. secalinus*	4×	11	5	5
*L. pseudoracemosus*	4×	10	6	7
*L. multicaulis*	4×	10	8	7
*L. racemosus*	4×	10	17	4
*L. coreanus*	4×	13	28	6
*L. arearius*	8×	22	0	12

The drawbacks to the conventional methods for detecting *P. huashanica* chromosomes (e.g., analyses involving Giemsa C-banding technology or molecular and biochemical markers) include the considerable time required to conduct the experiments and the production of low-resolution and inaccurate results. Wang et al. [34] characterized nine wheat–*P. huashanica* chromosome addition lines, with individual alien chromosomes identified according to Giemsa C-banding, but the distributed bands were unclear, which was detrimental for identifying chromosomes. By combining a GISH, Du et al. [67] identified a complete set of 1Ns–7Ns chromosomal addition lines using EST-SSR and EST-STS markers; however, the process was laborious and time-consuming. Biochemical markers, including HMW-GS, LMW-GS, and gliadin, are useful, but only for tracing the 1Ns and 6Ns chromosomes of *P. huashanica* [6]. FISH analyses involving suitable probes for identifying alien chromosomes are preferred by researchers because they are relatively simple to perform and inexpensive. For example, Du et al. [68] quickly and easily clarified seven CS–*Th. bessarabicum* alien chromosome introgressions by completing a FISH using *Th. bessarabicum*-specific oligo pools. Furthermore, 17 wheat–*A. cristatum* introgression lines containing different P-genome chromosomes were efficiently identified by using the isolated P-genome-specific DNA sequences for a FISH [69]. Li et al. [70] rapidly determined the chromosomal composition of eight wheat–tetraploid *Th. elongatum* hybrid derivatives using the pSc119.2, pTa535, pTa71, and pTa713 probe combination and suggested that the E-genome chromosomes of the tetraploid *Th. elongatum* can be quickly and accurately identified via FISH karyotyping in the wheat background. In our study, we efficiently characterized six wheat–*P. huashanica* chromosome addition lines in a FISH using pSc200, pTa71A-2, and Oligo-44. Our findings indicate that the *P. huashanica* Ns-genome chromosomes in the wheat background can be rapidly and precisely identified by a FISH using specific probes. These lines containing monosomic or diverse alien chromosomes have been selfed to obtain stable disomic chromosome addition lines. In future investigations, we will evaluate the agronomic performance and disease resistance of these lines and assess their utility as genetic resources relevant for wheat breeding.

## Conclusions

We constructed a FISH karyotype of *P. huashanica* using oligonucleotide probes pSc200, pTa71A-2 and Oligo-44. The combination of these probes helped distinguish all the Ns-genome chromosomes of *P. huashanica* (except for 4Ns) from those of wheat, diploid wheat relatives and most Ns-genome-containing species, providing an alternative for sequential GISH and FISH to detected *P. huashanica* chromosomes in the wheat background directly. Additionally, six wheat-*P. huashanica* addition lines were characterized and will be evaluated for agronomic performance and disease resistance. The FISH karyotype established here lays a solid foundation for the efficient identification of *P. huashanica* chromosomes in wheat genetic improvement programs.

## Data Availability

The datasets supporting the conclusions of this article are included within the article.
